# Supplementary material for: Pyruvate Dehydrogenase Kinase 1 inhibition mediated oxidative phosphorylation enhancement in cartilage promotes osteoarthritis progression
Source: BMC Musculoskelet Disord. 2023 Jul 20;24:597. doi: 10.1186/s12891-023-06585-6 (PMC10357736; doi:10.1186/s12891-023-06585-6)
Supplement: Supplementary file 1 — Additional file 1. [file 12891_2023_6585_MOESM1_ESM.zip › BP.Gsea.1653623667859/GOBP_POSITIVE_REGULATION_BY_HOST_OF_VIRAL_TRANSCRIPTION.html]

Details for gene set GOBP\_POSITIVE\_REGULATION\_BY\_HOST\_OF\_VIRAL\_TRANSCRIPTION[GSEA]

|  || Dataset | 55457\_collapsed\_to\_symbols.cls.cls #OA\_versus\_normal.cls.cls #OA\_versus\_normal\_repos |
| Phenotype | cls.cls#OA\_versus\_normal\_repos |
| Upregulated in class | normal |
| GeneSet | GOBP\_POSITIVE\_REGULATION\_BY\_HOST\_OF\_VIRAL\_TRANSCRIPTION |
| Enrichment Score (ES) | -0.64566505 |
| Normalized Enrichment Score (NES) | -1.8947442 |
| Nominal p-value | 0.0 |
| FDR q-value | 0.05404889 |
| FWER p-Value | 0.736 |
Table: GSEA Results Summary

  

Fig 1: Enrichment plot: GOBP\_POSITIVE\_REGULATION\_BY\_HOST\_OF\_VIRAL\_TRANSCRIPTION      
 Profile of the Running ES Score & Positions of GeneSet Members on the Rank Ordered List

  

| SYMBOL | TITLE | RANK IN GENE LIST | RANK METRIC SCORE | RUNNING ES | CORE ENRICHMENT || 1 | HPN | hepsin [Source:HGNC Symbol;Acc:HGNC:5155] | 4185 | 0.139 | -0.3053 | No |
| 2 | ZNF639 | zinc finger protein 639 [Source:HGNC Symbol;Acc:HGNC:30950] | 4274 | 0.131 | -0.2938 | No |
| 3 | SNW1 | SNW domain containing 1 [Source:HGNC Symbol;Acc:HGNC:16696] | 5472 | 0.035 | -0.3818 | No |
| 4 | TFAP4 | transcription factor AP-4 [Source:HGNC Symbol;Acc:HGNC:11745] | 6823 | -0.065 | -0.4775 | No |
| 5 | LEF1 | lymphoid enhancer binding factor 1 [Source:HGNC Symbol;Acc:HGNC:6551] | 7467 | -0.116 | -0.5112 | No |
| 6 | TAF11 | TATA-box binding protein associated factor 11 [Source:HGNC Symbol;Acc:HGNC:11544] | 8860 | -0.222 | -0.5882 | No |
| 7 | RRP1B | ribosomal RNA processing 1B [Source:HGNC Symbol;Acc:HGNC:23818] | 8909 | -0.225 | -0.5604 | No |
| 8 | SMARCA4 | "SWI/SNF related, matrix associated, actin dependent regulator of chromatin, subfamily a, member 4 [Source:HGNC Symbol;Acc:HGNC:11100]" | 10009 | -0.322 | -0.6005 | Yes |
| 9 | SMARCB1 | "SWI/SNF related, matrix associated, actin dependent regulator of chromatin, subfamily b, member 1 [Source:HGNC Symbol;Acc:HGNC:11103]" | 10117 | -0.333 | -0.5621 | Yes |
| 10 | CDK9 | cyclin dependent kinase 9 [Source:HGNC Symbol;Acc:HGNC:1780] | 11071 | -0.441 | -0.5743 | Yes |
| 11 | EP300 | E1A binding protein p300 [Source:HGNC Symbol;Acc:HGNC:3373] | 11741 | -0.540 | -0.5506 | Yes |
| 12 | CTDP1 | CTD phosphatase subunit 1 [Source:HGNC Symbol;Acc:HGNC:2498] | 11805 | -0.550 | -0.4784 | Yes |
| 13 | NUCKS1 | nuclear casein kinase and cyclin dependent kinase substrate 1 [Source:HGNC Symbol;Acc:HGNC:29923] | 11945 | -0.581 | -0.4077 | Yes |
| 14 | CCNT1 | cyclin T1 [Source:HGNC Symbol;Acc:HGNC:1599] | 12517 | -0.757 | -0.3460 | Yes |
| 15 | SP1 | Sp1 transcription factor [Source:HGNC Symbol;Acc:HGNC:11205] | 12557 | -0.777 | -0.2401 | Yes |
| 16 | CHD1 | chromodomain helicase DNA binding protein 1 [Source:HGNC Symbol;Acc:HGNC:1915] | 12745 | -0.908 | -0.1274 | Yes |
| 17 | JUN | "Jun proto-oncogene, AP-1 transcription factor subunit [Source:HGNC Symbol;Acc:HGNC:6204]" | 12813 | -0.994 | 0.0068 | Yes |
Table: GSEA details [plain text format]

  

Fig 2: GOBP\_POSITIVE\_REGULATION\_BY\_HOST\_OF\_VIRAL\_TRANSCRIPTION      
 Blue-Pink O' Gram in the Space of the Analyzed GeneSet

  

Fig 3: GOBP\_POSITIVE\_REGULATION\_BY\_HOST\_OF\_VIRAL\_TRANSCRIPTION: Random ES distribution      
 Gene set null distribution of ES for **GOBP\_POSITIVE\_REGULATION\_BY\_HOST\_OF\_VIRAL\_TRANSCRIPTION**
